# Supplementary material for: Public health impacts of an imminent Red Sea oil spill
Source: Nat Sustain. Author manuscript; Available in PMC 2021 Dec 17. (PMC8682806; doi:10.1038/s41893-021-00774-8)
Supplement: Supplementary Materials [file NIHMS1758071-supplement-Supplementary_Materials.pdf]

nature portfolio

https://doi.org/10.1038/s41893-021-00774-8

Supplementary information

Public health impacts of an imminent Red Sea oil spill

In the format provided by the authors and unedited

Supplementary Information: Public health impacts of an imminent Red Sea oil spill

Benjamin Q Huynh[1], Laura H Kwong[2], Mathew V Kiang[3,4], Elizabeth T Chin[1], Amir M Mohareb[5,6], Aisha O Jumaan[7], Sanjay Basu[8,9,10], Pascal Geldsetzer[11,12], Fatima M Karaki[*13], David H Rehkopf[*3]

1 Department of Biomedical Data Science, Stanford University School of Medicine, Stanford, CA, USA
2 Division of Environmental Health Sciences, School of Public Health, University of California, Berkeley, CA, USA
3 Department of Epidemiology and Population Health, Stanford University School of Medicine, Stanford, CA, USA
4 Harvard FXB Center for Health and Human Rights, Harvard University, Boston, MA, USA
5 Division of Infectious Diseases, Massachusetts General Hospital, Boston, MA, USA
6 Department of Medicine, Harvard Medical School. Boston, MA, USA
7 Yemen Relief and Reconstruction Foundation, Mercer Island, WA, USA
8 Center for Primary Care, Harvard Medical School, Boston, MA, USA
9 School of Public Health, Imperial College, London, UK
10 Institute of Health Policy, Management & Evaluation, University of Toronto, Toronto, ON
11 Division of Primary Care and Population Health, Department of Medicine, Stanford University School of Medicine, Stanford, CA, USA
12 Heidelberg Institute of Global Health, Heidelberg University, Heidelberg, Germany
13 Refugee and Asylum seeker Health Initiative, Department of Medicine, University of California San Francisco, San Francisco, CA, USA
*Contributed equally.

Correspondence: benhuynh@stanford.edu

Supplementary Analyses

Here we present additional sub-analyses showing how our estimates change when varying certain input parameters. Supplementary Figures 1, 2, 3, 4, and 5 are qualitatively similar to Figure 1, showing that varying spill duration (from 7 days to 24 hours), season (from winter and summer to autumn and spring), spatial resolution of spill particles (from 3 rounded decimal points of a longitude-latitude grid to 2), and number of spill particles (from 1000 to 10,000) do not substantially change our results in terms of simulated spills.

Supplementary Table 3 shows how varying the threshold of oil exposure moderately varies the amount of fisheries threatened by the oil spill. Supplementary Tables 4 shows increased hospitalization risk from air pollution using a different risk factor calculation, and Supplementary Table 5 shows increased mortality risk from air pollution.

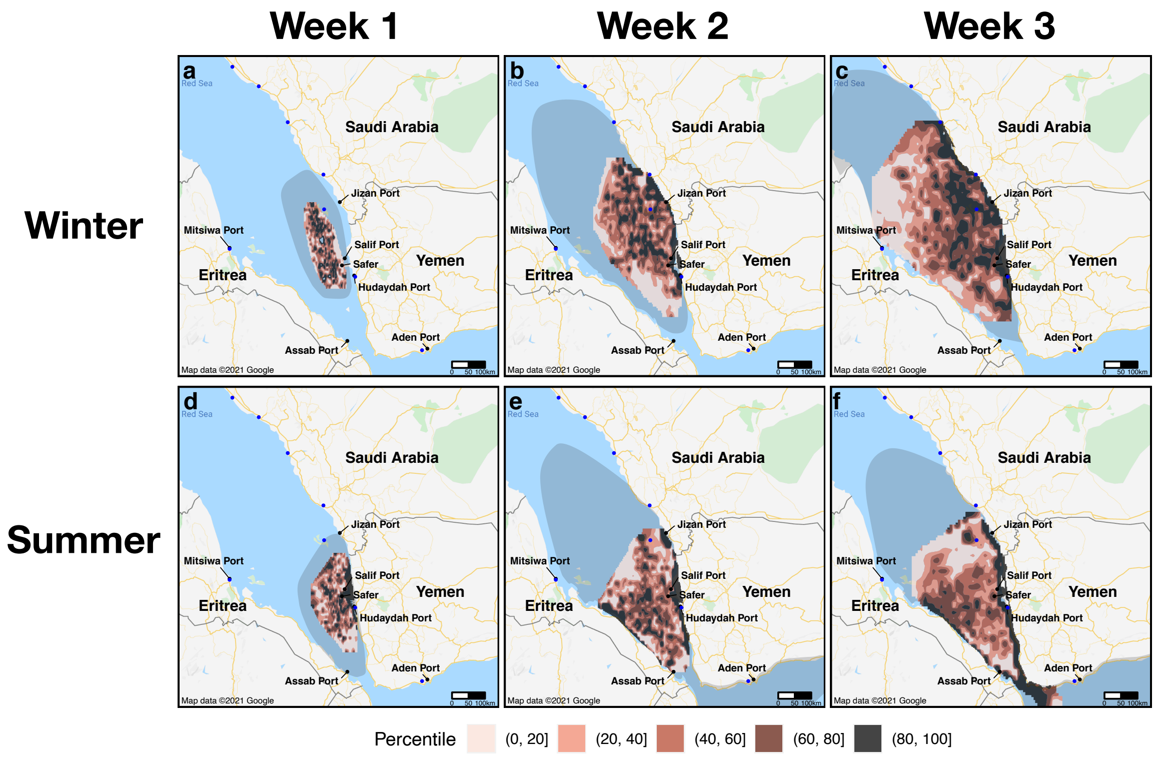

Supplementary Figure 1: Average surface oil concentration of 1000 simulated spills in the winter (a,b,c) and 1000 spills in the summer (d,e,f), assuming a 24-hour spill duration. Columns denote progress of the 1000 spills after one week (a,d), two weeks (b,e), and three weeks (c,f). Colored contours represent percentiles of average surface concentration over 1000 simulated spills. Shaded region represents the area within which approximately 90% of spill trajectories are expected to fall. Blue dots represent desalination plants.

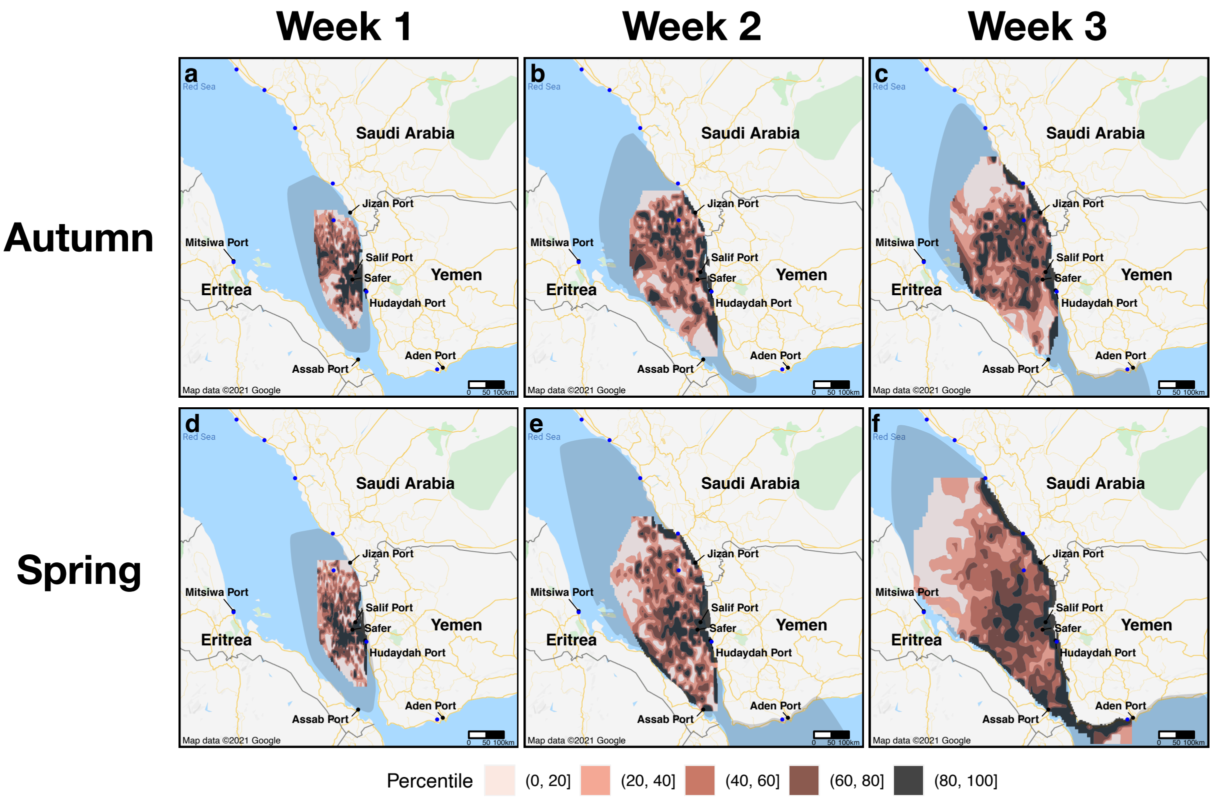

Supplementary Figure 2: Average surface oil concentration of 1000 simulated spills in the autumn (a,b,c) and 1000 spills in the spring (d,e,f). Columns denote progress of the 1000 spills after one week (a,d), two weeks (b,e), and three weeks (c,f). Colored contours represent percentiles of average surface concentration over 1000 simulated spills. Shaded region represents the area within which approximately 90% of spill trajectories are expected to fall. Blue dots represent desalination plants.

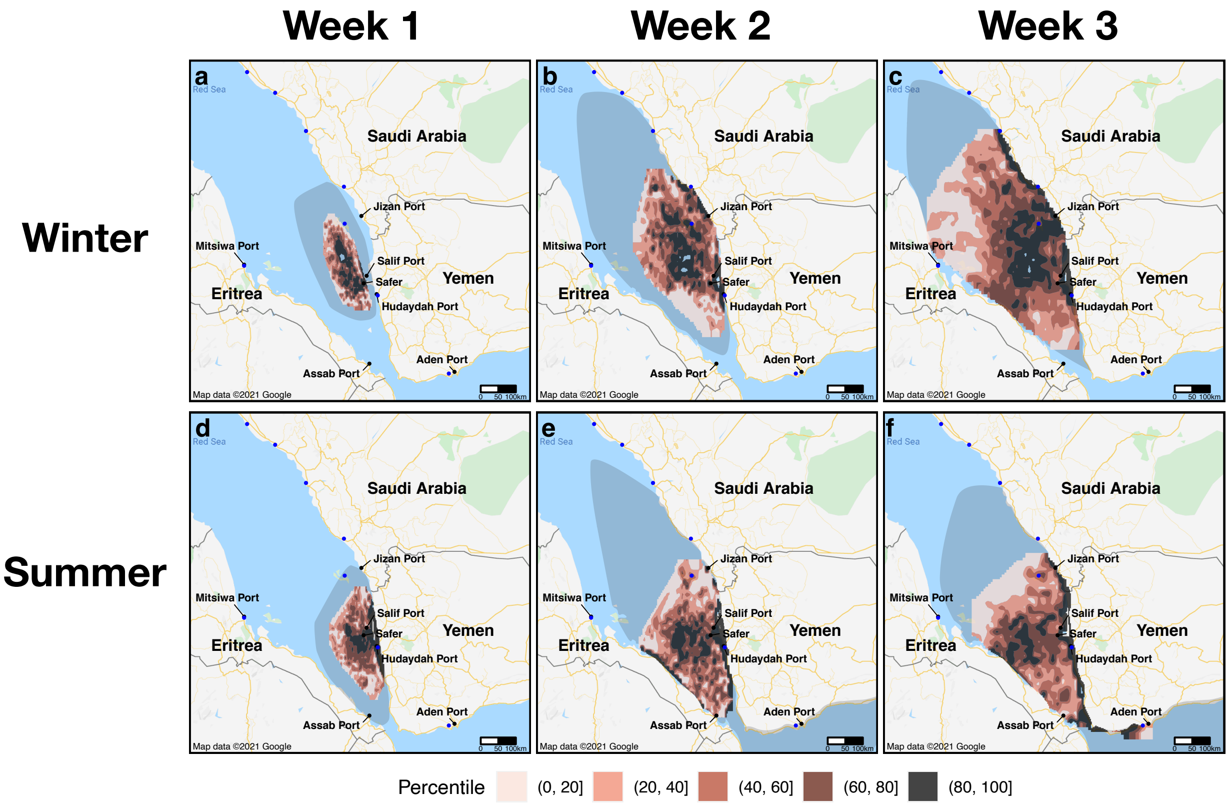

Supplementary Figure 3: Average surface oil concentration of 1000 simulated spills in the winter (a,b,c) and 1000 spills in the summer (d,e,f), with the spatial resolution rounded to two decimal points instead of three. Columns denote progress of the 1000 spills after one week (a,d), two weeks (b,e), and three weeks (c,f). Colored contours represent percentiles of average surface concentration over 1000 simulated spills. Shaded region represents the area within which approximately 90% of spill trajectories are expected to fall. Blue dots represent desalination plants.

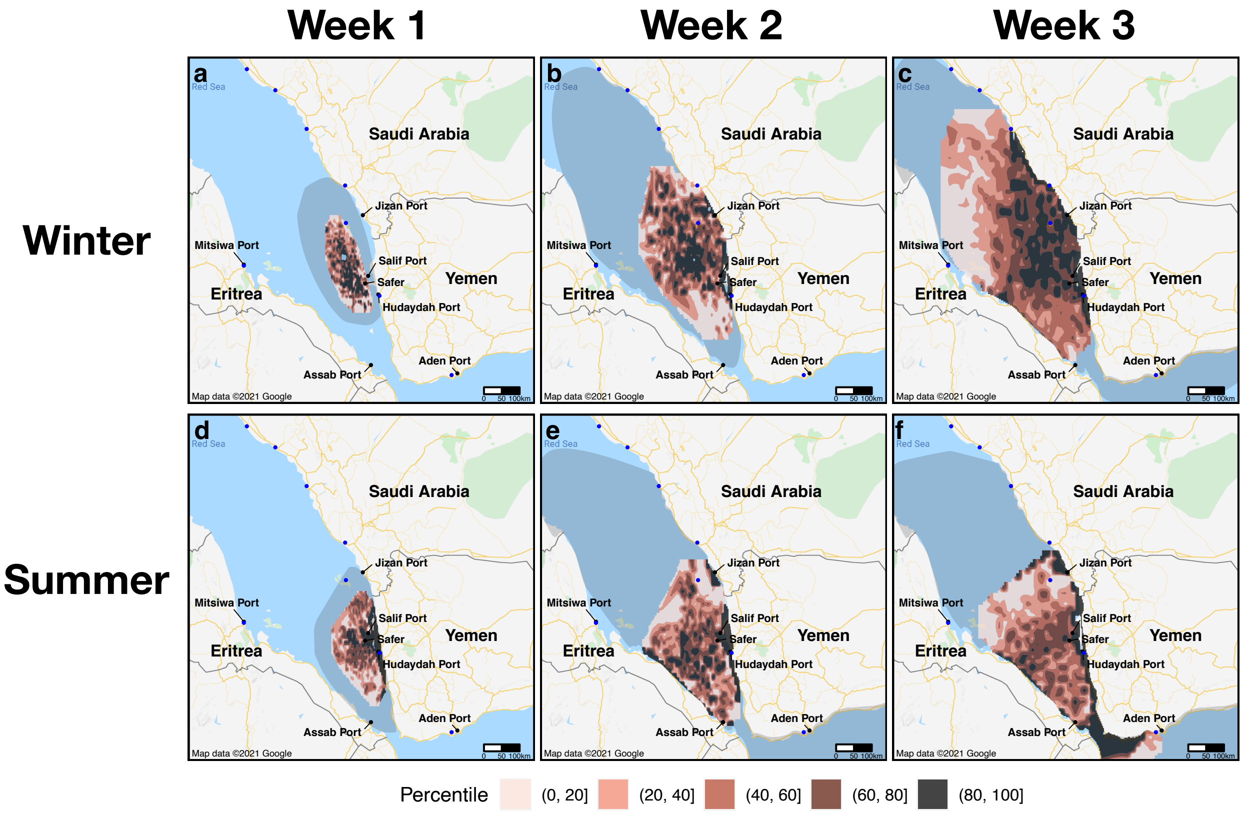

Supplementary Figure 4: Average surface oil concentration of 1000 simulated spills in the winter (a,b,c) and 1000 spills in the summer (d,e,f), with 10,000 particles per spill instead of 1000. Columns denote progress of the 1000 spills after one week (a,d), two weeks (b,e), and three weeks (c,f). Colored contours represent percentiles of average surface concentration over 1000 simulated spills. Shaded region represents the area within which approximately 90% of spill trajectories are expected to fall. Blue dots represent desalination plants.

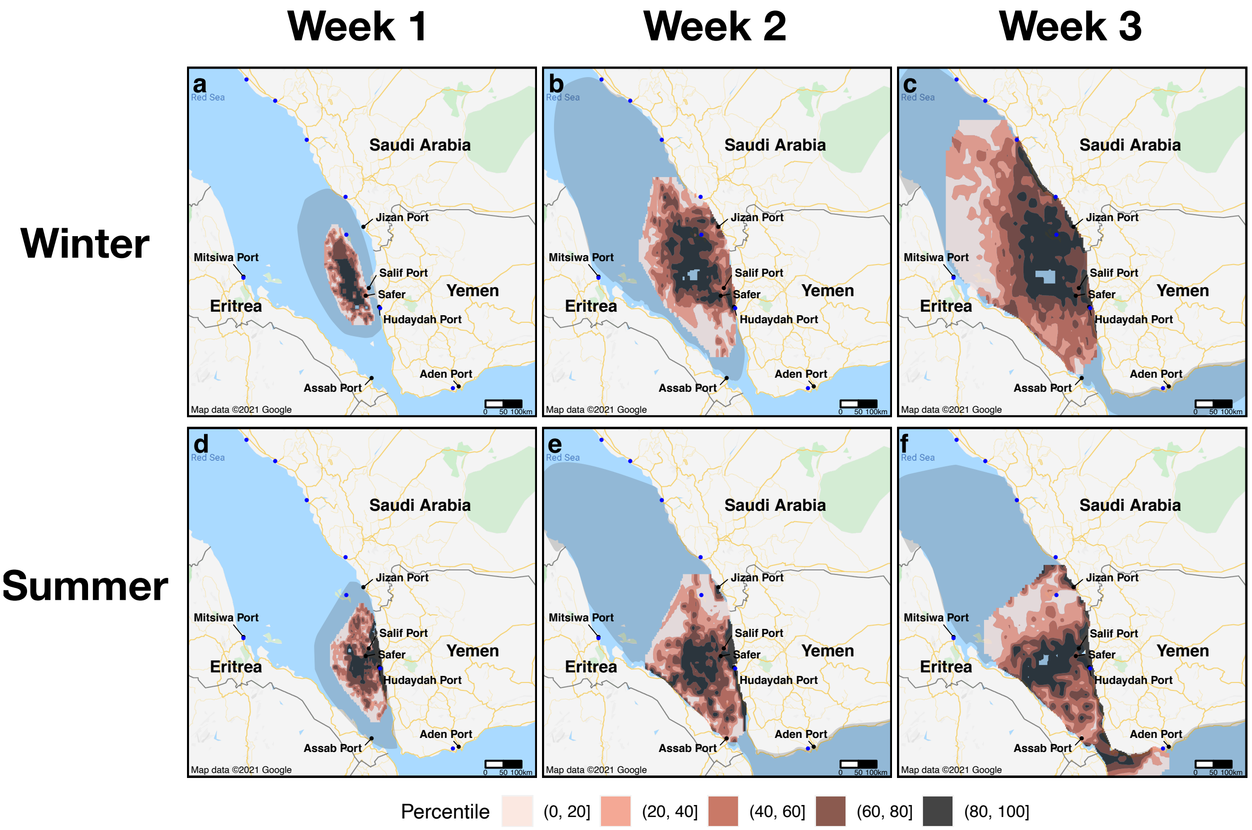

Supplementary Figure 5: Average surface oil concentration of 1000 simulated spills in the winter (a,b,c) and 1000 spills in the summer (d,e,f), with the spatial resolution rounded to two decimal points and 10,000 particles per spill. Columns denote progress of the 1000 spills after one week (a,d), two weeks (b,e), and three weeks (c,f). Colored contours represent percentiles of average surface concentration over 1000 simulated spills. Shaded region represents the area within which approximately 90% of spill trajectories are expected to fall. Blue dots represent desalination plants.

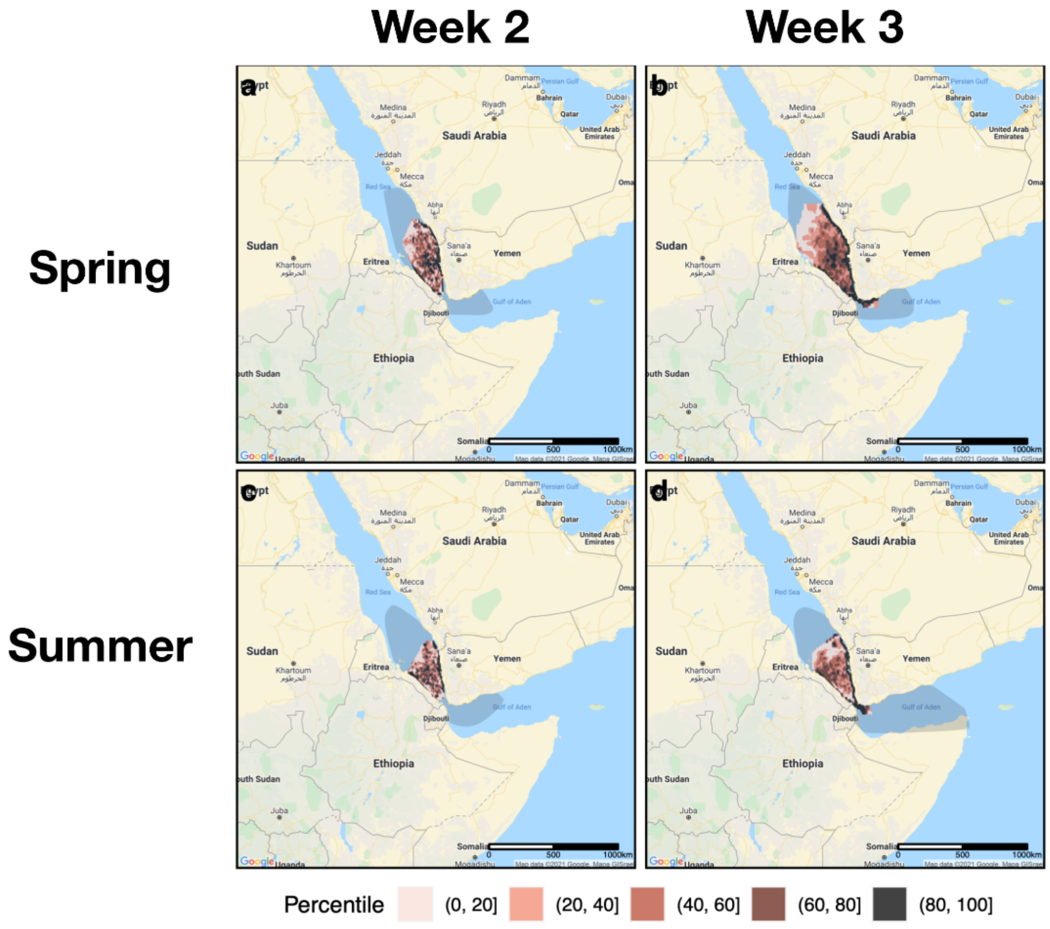

Supplementary Figure 6: Zoomed out view of oil spill simulations for Spring (a,b) and Summer (c,d) in weeks 2 (a,c) and 3 (b,d). Colored contours represent percentiles of average surface concentration over 1000 simulations. Shaded region represents the area within which approximately 90% of spill trajectories are expected to fall, which is now visible for the Gulf of Aden.

Tables

Supplementary Table 1: Percentiles of oil surface concentration amongst exposed areas averaged over simulations at locations of interest.

Location	Significance	Country	Week 1 Summer	Week 2 Summer	Week 3 Summer	Week 1 Winter	Week 2 Winter	Week 3 Winter
Hudaydah	Port	Yemen	94	96	94	0	98	97
Salif	Port	Yemen	84	90	88	0	91	94
Aden	Port	Yemen	0	0	26	0	0	0
Hudaydah	Desalination	Yemen	94	96	94	0	98	97

Aden	Desalination	Yemen	0	0	26	0	0	0
Assab	Port	Eritrea	0	23	24	0	0	0
Jizan	Port	Saudi Arabia	0	83	87	0	95	99
Farasan	Desalination	Saudi Arabia	0	55	66	0	65	70
Shuqaiq	Desalination	Saudi Arabia	0	0	0	0	0	95
Qunfudah	Desalination	Saudi Arabia	0	0	0	0	0	85

Supplementary Table 2: Locations and capacities of known desalination plants in the Southern Red Sea.

Desalination plant	Country	Coordinates	Capacity (m^3/day)
Hirgigo	Eritrea	15.580140, 39.449509	2,000
Farasan	Saudi Arabia	16.687464, 42.096350	9,000
Shuqaiq 1	Saudi Arabia	17.6599, 42.0761	94,000
Shuqaiq 2	Saudi Arabia	17.6599, 42.0761	216,000
Shuaiba	Saudi Arabia	20.675714, 39.528004	1,707,540
Qunfudah	Saudi Arabia	19.125537, 41.078224	9,000
Al Lith	Saudi Arabia	20.130047, 40.266668	9,000
Jeddah	Saudi Arabia	21.480555, 39.177983	663,532
Aden	Yemen	12.745462, 44.836638	34,000 (estimated)
Hudaydah	Yemen	14.800858, 42.966017	34,000 (estimated)

Supplementary Table 3: Estimated percent of total fish yield loss for Yemen in the Red Sea region by week after spill and season of spill. Different levels of oil exposure, measured in terms of percentiles of surface concentration relative to other exposed areas, are selected as minimum values to cause fish yield loss.

	Week 1	Week 2	Week 3
Winter, no threshold	74.6	87.86	97.96
Summer, no threshold	90.02	100	100
Winter, 10th percentile	66.47	87.86	92.96
Summer, 10th percentile	85.2	97.96	100
Winter, 20th percentile	64.34	80.03	92.96
Summer, 20th percentile	77.62	95.47	100

Supplementary table 4: Population-weighted average increased risk and exposed populations for respiratory hospitalizations from air pollution over various scenarios and spill durations. Spill duration is equivalent to exposure duration. All intervals denote 95% confidence intervals. Exposed population is defined as having been exposed to 10 ug/m3 or more of PM2.5. Uses estimates from Wei et al.[1]

Scenario	Average IR (%)	Affected population (person-days)
Summer, Fast-release, Leak	53.18 (50.4,55.96)	10968475
Winter, Fast-release, Leak	27.51 (26.15,28.87)	3117855
Summer, Slow-release, Leak	23.89 (22.73,25.05)	9190836
Winter, Slow-release, Leak	10.37 (9.89,10.85)	2318394
Summer, Fast-release, Fire	73.05 (69.56,76.54)	13247822
Winter, Fast-release, Fire	33.61 (32.07,35.14)	4256849
Summer, Slow-release, Fire	29.9 (28.51,31.29)	11761566

Winter, Slow-release, Fire	11.42 (10.9,11.93)	4078233

Supplementary table 5: Population-weighted average increased risk and exposed populations for cardiovascular and respiratory mortality from air pollution over various scenarios and spill durations. Spill duration is equivalent to exposure duration. All intervals denote 95% confidence intervals. Exposed population is defined as having been exposed to 10 ug/m3 or more of PM2.5. Uses estimates from Kloog et al.[2]

Scenario	Average IR (%)	Affected population (person-days)
Summer, Fast-release, Leak	26.73 (25.93,27.53)	10845335
Winter, Fast-release, Leak	13.55 (13.22,13.89)	3163042
Summer, Slow-release, Leak	11.75 (11.42,12.07)	9347517
Winter, Slow-release, Leak	5.04 (4.92,5.15)	2272926
Summer, Fast-release, Fire	34.98 (34.11,35.85)	13106374
Winter, Fast-release, Fire	16.68 (16.33,17.04)	4360401
Summer, Slow-release, Fire	15.27 (14.91,15.63)	11792403
Winter, Slow-release, Fire	5.64 (5.53,5.75)	4066740

Supplementary Table 6: Quantities reported in the main text in their rounded form and originally calculated form.

Quantity of interest	Reported estimate	Point estimate	95% UI (Reported)	95% UI (Calculated)
Oil evaporated after 24 hours	51	50.6	46-54	46.0275-53.9825

Monthly fuel disruption	200,000	206,723	180,000-250,000	184718.7-254009.7
Monthly food aid disruption (Hudaydah only)	5,700,000	5,684,800	3,700,000-8,100,000	3,695,800-8,122,600
Monthly food aid disruption (Aden included)	8,400,000	8,360,000	5,400,000-11,900,000	5,435,000-11,945,000
Increased risk of cardiovascular hospitalization at 1600ug/m3	530%	529%	460-590	464.2608-590.1955
